# RNAseq Analysis of Brown Adipose Tissue and Thyroid of Newborn Lambs Subjected to Short-Term Cold Exposure Reveals Signs of Early Whitening of Adipose Tissue

**DOI:** 10.3390/metabo12100996

**Published:** 2022-10-20

**Authors:** Andrea Graña-Baumgartner, Venkata S. R. Dukkipati, Paul R. Kenyon, Hugh T. Blair, Nicolás López-Villalobos, Kristene Gedye, Patrick J. Biggs

**Affiliations:** 1 School of Agriculture and Environment, Massey University, Private Bag 11 222, Palmerston North 4442, New Zealand; 2 School of Veterinary Science, Massey University, Private Bag 11 222, Palmerston North 4442, New Zealand; 3 School of Natural Sciences, Massey University, Private Bag 11 222, Palmerston North 4442, New Zealand

**Keywords:** RNAseq, BAT, thermogenesis, fat whitening, lambs

## Abstract

During the early postnatal period, lambs have the ability to thermoregulate body temperature via non-shivering thermogenesis through brown adipose tissue (BAT), which soon after birth begins to transform into white adipose tissue. An RNA seq approach was used to characterize the transcriptome of BAT and thyroid tissue in newborn lambs exposed to cold conditions. Fifteen newborn Romney lambs were selected and divided into three groups: group 1 (*n* = 3) was a control, and groups 2 and 3 (*n* = 6 each) were kept indoors for two days at an ambient temperature (20–22 °C) or at a cold temperature (4 °C), respectively. Sequencing was performed using a paired-end strategy through the BGISEQ-500 platform, followed by the identification of differentially expressed genes using DESeq2 and an enrichment analysis by g:Profiler. This study provides an in-depth expression network of the main characters involved in the thermogenesis and fat-whitening mechanisms that take place in the newborn lamb. Data revealed no significant differential expression of key thermogenic factors such as uncoupling protein 1, suggesting that the heat production peak under cold exposure might occur so rapidly and in such an immediate way that it may seem undetectable in BAT by day three of life. Moreover, these changes in expression might indicate the start of the whitening process of the adipose tissue, concluding the non-shivering thermogenesis period.

## 1. Introduction

In temperate countries such as New Zealand, where lambing occurs outdoors, newborn lambs transit rapidly from a warm uterine environment to a potentially harsh and cold external one. Under these conditions, lambs must immediately increase the rate of body heat production to fifteen times more than the fetal level to compensate for the increased heat loss [1,2]. As the ability to produce heat is essential for survival, lambs can thermoregulate their body temperature within minutes after birth, due to the presence of functional brown adipose tissue (BAT) [3]. This tissue, predominantly found in the peri-renal and pericardial areas, is the principal source of non-shivering thermogenesis in the newborn lamb [4,5], accounting for 60% of the generated heat [6]. The thermogenic activity of BAT is mainly regulated by catecholamines such as norepinephrine that are released from the sympathetic nervous system during cold exposure [7], which then activate β-adrenergic receptors expressed in the brown adipocytes [8]. This interaction results in a cascade of metabolic events that eventuate in the release of free fatty acids, which are the substrate for uncoupled oxidation and subsequently thermogenesis [9].

A BAT-specific transport protein of the inner mitochondrial membrane, uncoupling protein 1 (*UCP1*) or Thermogenin, supplies the thermogenic ability of BAT, and is activated by long-chain fatty acids produced by lipolysis upon adrenergic stimulation [10,11,12]. *UCP1* has the ability to increase proton permeability and form a proton-motive force through the mitochondrial matrix, where the energy produced from this force is then released as heat rather than stored as adenosine triphosphate (ATP) [9,13,14]. In addition, the thyroid hormone T3 (tri-iodothyronine) can act through its roughly 8000 receptors per brown adipocyte cell [15], stimulating the actions of *UCP1*, hence enhancing BAT thermogenesis [16,17]. In addition to this influence, thyroid hormone receptors act as a central inducer of BAT by mediating the synergism between thyroid hormone signalling and the sympathetic nervous system in BAT [18], increasing the capacity of cells to respond to catecholamines [19,20], and thereby increasing adrenergic sensitivity [21]. These findings demonstrate the multifaceted contribution to thermogenesis involving the crucial relationship between thyroid hormones and BAT, and the importance of their joint contribution to energy homoeostasis [16].

The secretion and plasma concentrations of many of these above-mentioned factors decline over the first few days and weeks of a lamb’s life [22,23], as shivering thermogenesis becomes the main response to cold exposure, replacing the non-shivering mechanisms of heat production [4,24]. This replacement has an evolutionary implication, since although BAT is an efficient heat producer, it is small and localized, so the heat it produces needs to be circularized through the body to maintain the core body temperature [25]. As opposed to this, skeletal muscle is dispersed throughout the body, making it possible to produce heat in situ in many parts of the body at the same time [25]. Consequently, heat produced from skeletal muscle shivering is more cost-effective than BAT; thus, it reduces the demands on diet intake, giving the possibility to invade and explore new geographic areas that are cooler and where food availability is low [26].

At birth, *UCP1* is at its maximum expression but this decreases rapidly, and by day four of life it seems to become nearly undetectable, as observed by Basse et al. [3]. Consequently, brown adipocyte formation and BAT’s thermogenic activity also decrease, as BAT begins its transition into white adipose tissue (WAT) which becomes predominant by day 30 [27,28,29]. WAT then only acts as the main fat storage of the body, losing the large mitochondrial phenotype of BAT and the expression of its related genes involved in heat production [3,6,8,28].

Studying the molecular components of heat production, and the signals exchanged between BAT and the thyroid glands is crucial to help the understanding of the underlying regulatory mechanisms that control thermogenesis in newborn lambs. No published reports appear to have used a ribonucleic acid sequencing (RNAseq) approach in BAT and thyroid glands tissue, to explore the newborn lamb metabolic activity in response to cold exposure. For that reason, the objective of this study was to characterize the transcriptome of brown adipose tissue and thyroid tissue through RNAseq in newborn Romney lambs exposed to either 22 °C or 4 °C for 2 days.

## 2. Materials and Methods

This study was undertaken in spring 2018 at the Massey University Animal Physiology Unit (APU) with newborn Romney-type lambs born on Keebles Farm (40°24′ S, 175°36′ E), Palmerston North, New Zealand.

### 2.1. Animals and Sampling

During the lambing period, which was outdoors under pastoral conditions, fifteen newborn Romney lambs (9 males and 6 females) were randomly procured. Lambs were born as twins, but only one lamb per dam was used (the heaviest of the set). Within 12–24 h after birth, the selected lamb was tagged and the ewe together with both of its lambs were brought indoors to the APU. The selected lamb within each twin set was then weighed and allocated randomly to one of the three treatment groups: group 1 (*n* = 3; two males weighing 4.8 kg and 5.2 kg, respectively, and a female weighing 4.4 kg) as a control; group 2 (*n* = 6; two females weighing 4.9 and 4.5 kg, respectively, and four males weighing 4.6, 6.0, 4.6 and 5.3 kg, respectively), which were kept indoors for two days at an ambient temperature (20–22 °C); and group 3 (*n* = 6; two females weighing 5.5 and 6.1 kg, respectively, and four males weighing 5.4, 4.5, 5.4 and 5.2 kg, respectively), which were kept indoors for two days at a cold temperature (4 °C). This followed the previously described method of Marcher et al. [30] to induce a cold challenge. Soon after being brought to the APU, the three lambs in group 1 were euthanized via captive bolt to provide a baseline transcriptome profile. Samples of brown adipose tissue (BAT) from around the kidneys and the thyroid tissue were collected and stored at −80 °C. Lambs in groups 2 and 3, together with their dams and siblings were moved into indoor pens (2 m by 1 m) for two days. Siblings of the lambs in group 3, which were not subjects of the current study, were wrapped with wool covers (Woolover Limited, Christchurch, New Zealand) to enhance their comfort and to minimize impact of the cold. During the two days of indoor retention, the ewes were fed commercial roughage (FiberEzy, Fiber Fresh Feeds Ltd., Reporoa, New Zealand) and commercial grain-based pellets (10%) (NRM Sheep Nuts, Northern Roller Mills, Christchurch, New Zealand) and had unrestricted access to water. The ewes and lambs were monitored at least 3 times per day during this period, to ensure successful ewe/lamb bonding and that the lambs were successfully suckling the ewe. On day 2, after 48 h exposure at respective temperatures, the 12 lambs of these two groups (i.e., 6 per group) were euthanized by captive bolt. Samples of brown adipose tissue from around the kidneys and the thyroid tissue were collected and stored at −80 °C. In all groups, after the lambs had been euthanized, their dams and remaining siblings were returned to Keebles Farm to commercial farming conditions.

### 2.2. RNA Isolation and Transcriptome Sequencing

Brown adipose tissue from around the kidneys and thyroid tissue samples were submitted to BGI Genomics (Hong Kong) for RNA extraction and subsequent sequencing. Approximately 60 mg of tissue per sample was ground with liquid nitrogen into powder, then transferred into a 2 mL tube containing 1.5 mL of TRIzol (Invitrogen, Carlsbad, CA, USA). The samples were homogenized with the TissueLyser II (Qiagen, Hilden, Germany), and then incubated at room temperature for 5 min to permit the complete dissociation of nucleoprotein complexes, before being centrifuged at 12,000× *g* for 5 min at 4 °C (Centrifuge 5427R, Eppendorf, Hamburg, Germany). The resulting supernatant was transferred into a new 2 mL tube, 0.3 mL of chloroform/isoamyl alcohol (24:1) was added, and then mixed by vigorously shaking the tubes for 15 s. The samples were then centrifuged at 12,000× *g* for 10 min at 4 °C, to separate the mixture into three layers; the lower phenol-chloroform phase, an interphase and the upper aqueous phase containing the RNA. The aqueous phase was transferred into a new 1.5 mL tube and an equal volume of isopropyl alcohol was added; this was mixed and incubated at −20 °C for 2 h for precipitation. After this time, the samples were centrifuged at 13,600× *g* for 20 min at 4 °C. Following this centrifugation, the supernatant was removed, and the RNA pellet was washed with 1 mL of 75% ethanol then resuspended and centrifuged at 13,600× *g* for 3 min at 4 °C. This step was repeated, and at the end the ethanol was removed without disturbing the pellet, which was let to air-dry in a biosafety cabinet (Esco Airstream, Esco Technologies, Horsham, PA, USA). Afterwards, 25–100 µL of diethyl pyrocarbonate-treated water was added to solubilize the RNA pellet.

The quality check of the extracted RNA was measured by RNA integrity number (RIN), where on average, a RIN of 7.8 for thyroid tissue and 6.8 for brown adipose tissue was observed (Table S1). Following the RNA isolation and quality checks, mRNA was purified from total RNA using oligo (dT)-attached magnetic beads. The resulting mRNA molecules were fragmented and reverse-transcribed into double-stranded cDNA by using random hexamer primers. The synthesized cDNA was subjected to an end repair and then 3′ adenylated, where adapters were ligated to the ends of these fragments. The cDNA fragments were amplified by PCR and product purified with Agencourt AMPure XP beads (Beckman Coulter, Brea, CA, USA). Afterwards, a quality check was conducted on an Agilent 2100 Bioanalyzer (Agilent Technologies, Santa Clara, CA, USA). The PCR product was then heat-denatured and the single-strand DNA was circularized by splint oligo and DNA ligase. Finally, all cDNA libraries were sequenced using paired-end strategy of 150 bp read length, through the BGISEQ-500 platform.

### 2.3. Quality Control, Mapping and Reads Quantification

All processes described in this section were carried out through the use of high-performance computing resources of the New Zealand eScience Infrastructure (NeSI, https://www.nesi.org.nz/).

The quality of the raw 150-base paired-end sequence files was examined using FastQC (version 0.11.9) (https://www.bioinformatics.babraham.ac.uk/projects/fastqc/) and then summarized by MultiQC (version 1.9) as described by Ewels et al. [31]. Next, the paired-end reads were trimmed to remove low-quality bases and adapter sequences with Trimmomatic (version 0.39) [32], using the options: SLIDINGWINDOW:4:20, MINLEN:50, ILLUMINACLIP:TruSeq3-PE.fa:2:30:10. The read quality was re-assessed with FastQC and MultiQC to confirm that improvements had been made by trimming. Clean reads were mapped to a reference genome (Oar_rambouillet_v1.0) from the RefSeq database (https://www.ncbi.nlm.nih.gov/assembly/GCF_002742125.1/ accessed on 22 April 2021) using STAR (version 2.7.7a) [33]. After mapping, featureCounts (Subread package, version 2.0.0) was used to quantify the aligned reads [34], allowing multimapping reads to be counted using the option -M.

Principal component analysis (PCA) plots of normalised sequence reads were constructed in R (version 4.1.0) [35], inside RStudio (version 1.10.0) [36] with the “ggplot2” package (version 3.3.5) [37], to visualize the differences between control, cold and ambient temperature samples for BAT and thyroid tissue, according to the top 1000 genes per tissue selected by highest row variance (sample variance). Heatmaps of the normalized counts per gene, for the 38 analyzed genes, from the DESeq2 analysis for each group of samples (control, cold, and ambient) were made in R (version 4.1.0) [35], inside RStudio (version 1.10.0) [36] with the “pheatmap” package (version 1.0.12) [38], and the“ RColorBrewer” package (version 1.1-2) [39]. The genes *ADRB3* and *THRB* were excluded from the thyroid tissue heatmap due to insufficient counts.

### 2.4. Differential Expression Analysis

Differentially expressed genes (DEGs) were identified using the DESeq2 package (version 1.32.0) [40], in R (version 4.1.0) [35], inside RStudio (version 1.10.0) [36]. Low read counts were prefiltered before running DESeq2, by removing rows that had fewer than 10 reads in at least 10 samples. Each tissue (BAT and thyroid) was analyzed separately and the DEGs were examined by treatment vs. control (ambient temperature vs. control, and cold temperature vs. control), in order to compare the differences in the transcriptome after being exposed to different temperatures for two days vs. not being exposed to them. For normalization, three selected reference genes were utilized: *ACTB*, *RPL19* and *HUWE1,* as they were previously tested [41,42,43]. For both tissues, one control sample was removed as it had outlier counts overall, compared to all the other samples. Finally, genes with an absolute value of |log2Fold Change| ≥ 1 and an adjusted *p*-value < 0.05 were considered differentially expressed and were used in subsequent analyses (Table S2). The adjusted *p*-value was calculated using the Benjamini–Hochberg False Discovery Rate (FDR) concept [44]. In addition to the treatment vs. control analysis, an analysis of cold temperature vs. ambient temperature for each tissue was also performed in a unique set of 38 genes that, according to the literature, are involved in thermogenesis or fat whitening. These were: *UCP1*, *ADRB1*, *ADRB2*, *ADRB3*, *ADRA1A*, *PPARGC1A*, *PPARGC1B*, *PPARA*, *PPARG*, *ELOVL6*, *BMP4*, *BMP7*, *BMP8B*, *CIDEA*, *CKB*, *PDK4*, *TGM2*, *FNDC5*, *ACSL5*, *CPT1A*, *FABP3*, *PRKG1*, *NOS3*, *PDE3B*, *VASP*, *LPL*, *PRDM16*, *EHMT1*, *GABPA*, *VEGFA*, *CYP1A1*, *THRA*, *THRB*, *DIO2*, *PNPLA2*, *LIPE*, *MGLL* and *MKI67* (Table S3). Plots depicting the intersection of the number of genes between experimental groups were constructed in R (version 4.1.0) [35], inside RStudio (version 1.10.0) [36] with the “tidyverse” package (version 1.3.1) [45] and the “UpSetR” package (version 1.4.0) [46]. Volcano plots of the results from each tissue (showing treatment vs. control) were created using the“ EnhancedVolcano” package (version 1.14.0) [47] in R (version 4.1.0) [35], inside RStudio (version 1.10.0) [36].

### 2.5. Functional Analysis of DEGs

Gene ontology (GO) enrichment analysis, Reactome pathway database and Kyoto Encyclopedia of Genes and Genomes (KEGG) pathways were performed in g:Profiler (https://biit.cs.ut.ee/gprofiler/gost (accessed on 6 September 2021)) [48,49,50], as it has previously been utilized in RNAseq analysis [51,52,53]. Analysis was conducted through an ordered query with a significance threshold by the Benjamini–Hochberg FDR method (adjusted *p*-value < 0.05) (Table S4). A human database was set for all analyses, due to the scarcity of sheep GO data [54]. Additional information regarding selected genes of interest was gathered through the NCBI database (https://www.ncbi.nlm.nih.gov/gene/).

### 2.6. Validation by Reverse Transcription-Quantitative Polymerase Chain Reaction (RT-qPCR)

Total RNA from BAT and thyroid tissue of all 15 lambs (3 of group 1, 6 of group 2 and 6 of group 3) was extracted using TRIzol (Invitrogen, Carlsbad, CA, USA). Approximately 50–60 mg of tissue was homogenized with 1 mL of TRIzol and around 50 mg of a mixture of 1.0:2.3 zirconia/silica beads (dnature, Gisborne, New Zealand), in a TissueLyser II (Qiagen, Hilden, Germany) with 30 Hz frequency. This homogenization was performed for 2 min for the BAT samples and 5 min for the thyroid samples, all conducted in intervals of 30 s with 1 min of in-ice storage in between. After homogenization, the samples were incubated at room temperature for 5 min. Subsequently, a centrifugation step at 12,000× *g* at 4 °C for 10 min was performed and the supernatant was pipetted carefully into a clean 2 mL tube. To the supernatant, 200 µL of chloroform was then added and mixed by vortexing; after this, the sample was incubated for 5 min at room temperature. The sample was then centrifuged at 12,000× *g* at 4 °C for 20 min and the upper aqueous RNA phase was transferred into a new 2 mL tube without disturbing the interphase layer. The volume of the sample was estimated, and 1.5 volumes of 100% ethanol were added then mixed by inverting the tube several times. RNA was extracted from the ethanol-precipitated samples using RNeasy Mini Kit (Qiagen, Hilden, Germany), following the kit protocol. Final RNA was resuspended in 33 μL of RNAse-free water. The concentration of RNA was determined by measuring the absorbance at 260 nm and its purity evaluated at an absorption ratio of 260/280 nm, using a NanoDrop spectrophotometer (Thermo Scientific, MA, USA). The RNA was stored at −80 °C until RT-qPCR reactions were performed.

The expression levels of six selected DEGs were determined relative to *ACTB* and *GAPDH* endogenous control genes, which remained unchanged among samples in this study. Specific primers (Table S5) for *ACTB*, *KI67*, *BMP4*, *DIO2* and *PPARGC1A* were designed using Primer3, with gene sequences extracted from the reference genome described previously, through Geneious software (version 10.2.6). The primer sequences for *GAPDH*, *VEGFA* and *ADRB3* were based on previous sheep-based studies [29,55,56]. Single-step RT-qPCRs were performed using Verso 1-step RT-qPCR Kit (Thermo Fisher Scientific, MA, USA). Each 20 µL reaction mix included: 10 µL Verso 1 Step qPCR SYBR Mix, 1 µL Verso RT Enhancer, 0.2 µL Verso Enzyme Mix, 5 µL RNase/DNase-free water, 1.4 µL each of the forward and reverse primers (final concentration 700nM) (Integrated DNA Technologies, Coralville, USA) and 1 µL (containing approximately 2.20 ng) of diluted RNA. Reactions were set up in triplicate in a 36-disk Rotor-Gene Q RT-PCR cycler (Qiagen). The standard amplification conditions included: cDNA synthesis at 50 °C for 15 min, an initial denaturation of the cDNA and enzyme activation at 95 °C for 15 min, followed by 40 cycles of 30 s at 95 °C, 30 s at the appropriate annealing temperature (60 °C for *ACTB* and *GAPDH*, 55 °C for all other primer combinations), and 30 s at 72 °C with fluorescence capture. At the end of each run, dissociation curves (from 72 °C to 95 °C with a fluorescent absorbance reading after each 0.3 °C increment) were analyzed to ensure that the desired amplicon was being detected and to discard contaminating DNA or primer dimers. Expression data were generated using the mathematical model of 2^−ΔΔCT^ [57] and normalized to the geometric mean expression of the endogenous control genes.

## 3. Results

### 3.1. Summary of Sequencing Reads

On average from BGI sequencing, each BAT and TH sample had 73,597,270 and 73,552,149 reads, respectively. After quality control analysis, on average, 55,337,991 (75.28%) and 37,777,815 (51.36%) reads in BAT and TH, respectively, were mapped to the reference genome (Oar_rambouillet_v1.0) through the STAR software. From those, 33,953,923 (61.49%), and 27,886.474 (73.98%) reads, respectively, in BAT and TH were assigned to features through the featureCounts software. In summary, an average of 46.13% of reads in BAT and 37.91% in TH were assigned to features from the original sequencing reads. A detailed version on reads per sample throughout the analysis is presented in Supplementary Table S1. The PCA of normalized sequence reads revealed a separation of the three groups in BAT as well as thyroid (Figure S1).

### 3.2. Summary of DEGs

**Figure 1:**
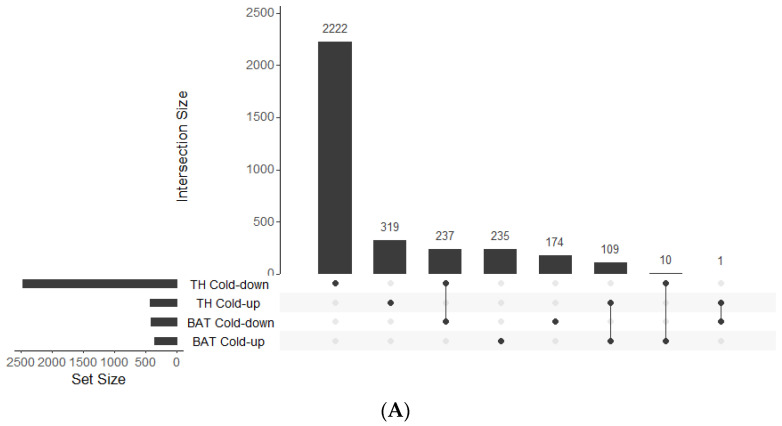

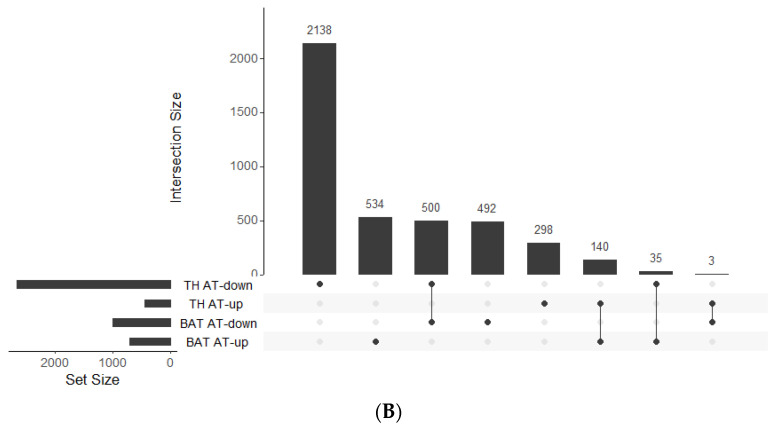
UpSet plots showing the intersection of the number of genes between experimental groups. (**A**) Intersection of genes expressed in BAT and thyroid tissue (TH) of lambs under cold temperature (4 °C). (**B**) Intersection of genes expressed in BAT and thyroid tissue of lambs under ambient temperature (AT, 20–22 °C). All genes depicted here had a cutoff of log2Fold Change ≥ |1| and an adjusted *p*-value < 0.05. The UpSet plots visualize the intersection between sets, which are the rows in the matrix, and the columns correspond to the intersections between these sets.

**Figure 2:**
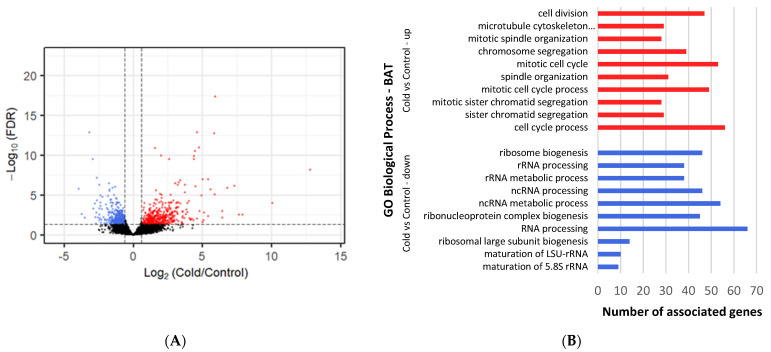
Differential gene expression and enrichment in BAT of lambs under cold temperature (4 °C). (**A**) Volcano plot comparing downregulated (blue dots) and upregulated (red dots) gene expression that occurs after cold exposure. Dotted lines indicate cutoffs, *p* adj-value < 0.05 and log2Fold Change (Cold/Control) ≥ |1|. Black dots represent genes that are not significantly different. (**B**) The most significant (those with lowest *p*-value) up (red lines) and downregulated (blue lines) DEGs enriched Biological Process GO terms, as determined by g:Profiler (*p* adj-value < 0.05), in brown fat tissue exposed to cold. Names of GO terms are indicated on the Y-axis, and the number of enriched genes for each term is represented on the X-axis.

**Figure 3:**
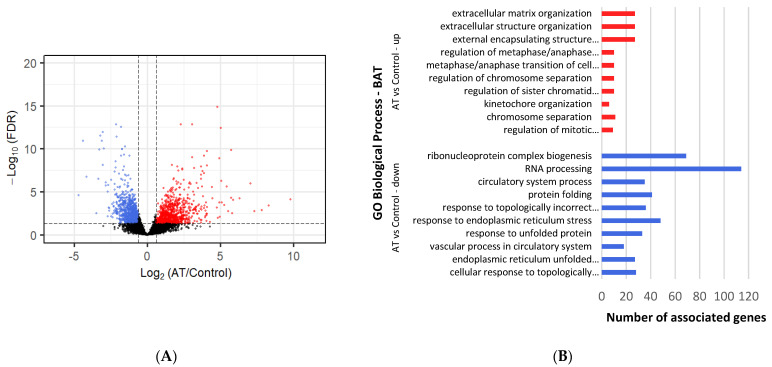
Differential gene expression and enrichment in BAT of lambs under ambient temperature (20–22 °C). (**A**) Volcano plot comparing downregulated (blue dots) and upregulated (red dots) gene expression that occurs at AT exposure. Dotted lines indicate cutoffs, *p* adj-value < 0.05 and log2Fold Change (AT/Control) ≥ |1|. Black dots represent genes that are not significantly different. (**B**) The most significant (those with lowest *p*-value) up (red lines) and downregulated (blue lines) DEGs enriched Biological Process GO terms, as determined by g:Profiler (*p* adj-value < 0.05), in brown fat tissue at AT. Names of GO terms are indicated on the Y-axis, and the number of enriched genes for each term is represented on the X-axis.

**Figure 4:**
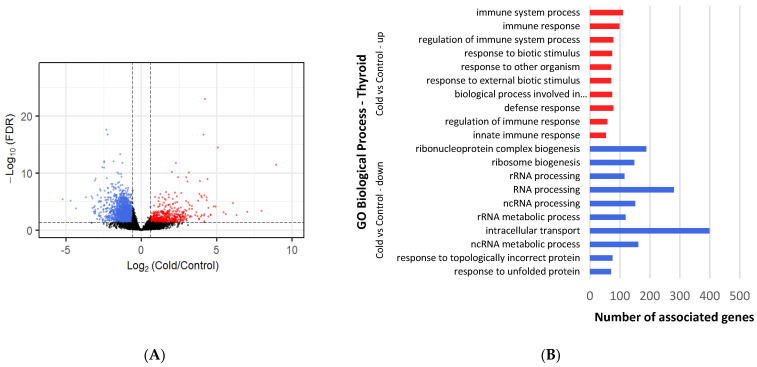
Differential gene expression and enrichment in thyroid tissue of lambs under cold temperature (4 °C). (**A**) Volcano plot comparing downregulated (blue dots) and upregulated (red dots) gene expression that occurs after cold exposure. Dotted lines indicate cutoffs, *p* adj-value < 0.05 and log2Fold Change (cold/control) ≥ |1|. Black dots represent genes that are not significantly different. (**B**) The most significant (those with lowest *p*-value) up (red lines) and downregulated (blue lines) DEGs enriched Biological Process GO terms, as determined by g:Profiler (*p* adj-value < 0.05), in thyroid tissue exposed to cold. Names of GO terms are indicated on the Y-axis, and the number of enriched genes for each term is represented on the X-axis.

**Figure 5:**
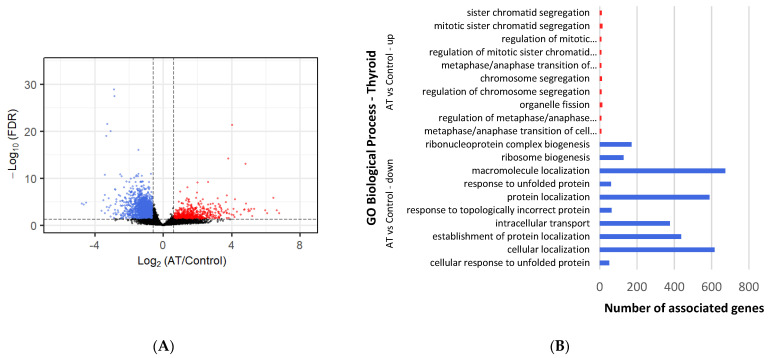
Differential gene expression and enrichment in thyroid tissue of lambs under ambient temperature (20–22 °C). (**A**) Volcano plot comparing downregulated (blue dots) and upregulated (red dots) gene expression that occurs at AT exposure. Dotted lines indicate cutoffs, *p* adj-value < 0.05 and log2Fold Change (AT/Control) ≥ |1|. Black dots represent genes that are not significantly different. (**B**) The most significant (those with lowest *p*-value) up (red lines) and downregulated (blue lines) DEGs enriched Biological Process GO terms, as determined by g:Profiler (*p* adj-value < 0.05), in thyroid tissue at AT. Names of GO terms are indicated on the Y-axis, and the number of enriched genes for each term is represented on the X-axis.

All DEGs with a cutoff of log2Fold Change ≥ |1| and an adjusted *p*-value < 0.05 (Table S2) were selected for comparison analysis against controls (Figure 1). Under cold conditions, 354 genes were found to be upregulated and 412 downregulated in BAT (Figure 2A), whereas 709 upregulated and 995 downregulated genes were identified under ambient conditions (Figure 3A). Under cold conditions, 429 upregulated and 2469 downregulated genes were detected in thyroid tissue (Figure 4A), whereas 441 upregulated and 2673 downregulated genes were found under ambient conditions (Figure 5A).

### 3.3. Differential Gene Expression and Enrichment in BAT under Cold Conditions

A total of 638 upregulated Biological Process (BP) terms were found (Table S4). The most significant ones (those with the lowest *p*-value) were involved in the cell cycle with terms such as “cell division”, “chromosome segregation” and “mitotic cell cycle” (Figure 2B). Moreover, there were a considerable number of terms regarding immune defense, such as “response to virus” (26 genes), “immune system process” (33 genes) and “defense response” (24 genes). Stress-related BP terms were also found: “response to stress” (35 genes) and “regulation of response to stress” (17 genes). In addition, “response to stimulus” terms appear significant, such as “regulation of response to external stimulus” (34 genes) and “cellular response to stimulus” (55 genes). Further, cell communication and signaling terms appeared, such as “positive regulation of signaling” (31 genes), “cell surface receptor signaling pathway” (17 genes) and “regulation of signaling” (55 genes). For the Molecular Function (MF) category, “microtubule binding” (20 genes), “extracellular matrix structural constituent” (13 genes) and “ATPase” (19 genes) were found as being the most significant. Within the Cellular Component category (CC) “chromosome-centromeric region” (24 genes), “extracellular matrix” (31 genes) and “cell periphery” (97 genes) were part of the most significant terms. The top Reactome pathways were “arachidonic acid metabolism” (3 genes) and “fatty acid metabolism” (4 genes).

A total of 544 downregulated BP terms were found (Table S4). These terms were focused on ribosome synthesis and maturation, with “ribosome biogenesis”, “rRNA processing” and “RNA processing” as the most significant ones (Figure 2B). In addition, many terms associated with protein synthesis and maturation were found: “protein folding” (21 genes), “positive regulation of cellular protein localization” (18 genes) and “protein maturation” (16 genes). In the MF category, the most enriched terms were “RNA binding” (101 genes), “unfolded protein binding” (13 genes) and “ribonucleoprotein complex binding” (12 genes). The most enriched terms for CC included “preribosome” (23 genes), “nucleolus” (53 genes) and “ribonucleoprotein complex” (47 genes). Further, the most significant pathway for KEGG analysis was “Ribosome biogenesis in eukaryotes” (15 genes) and for Reactome analysis it was “rRNA processing” (26 genes).

### 3.4. Differential Gene Expression and Enrichment in BAT under Ambient-Temperature Conditions

A total of 40 upregulated BP terms were found (Table S4). The most significant ones were focused on cell cycle and the extracellular space, with terms such as “extracellular matrix organization”, “regulation of metaphase/anaphase transition of cell cycle” and “regulation of chromosome separation” (Figure 3B). These were followed closely by immune system terms, such as “antiviral innate immune response” (3 genes), “type I interferon signaling pathway” (9 genes) and “response to virus” (5 genes). Within the MF category, “aldehyde dehydrogenase (NAD+) activity” (7 genes), “extracellular matrix structural constituent” (16 genes) and “integrin binding” (13 genes) were found as most significant. The most significant CC terms were “extracellular matrix” (39 genes), “cell periphery” (159 genes) and “chromosome, centromeric region” (16 genes). The term “interferon alpha/beta signaling” (4 genes) was seen as the most significant in Reactome pathways.

A total of 187 downregulated BP terms were found (Table S4). The most significant terms were linked to RNA and protein processing, such as “ribonucleoprotein complex biogenesis”, “RNA processing” and “protein folding” (Figure 3B). Further, stress-related BP terms appeared as well, such as: “cellular response to stress” (168 genes) and “regulation of cellular response to stress” (69 genes). The most significant MF terms were “RNA binding” (180 genes), “enzyme binding” (166 genes) and “protein binding” (846 genes). Intracellular-related terms were found to be the most significant type of CC terms, including: “cytoplasm” (739 genes), “intracellular membrane-bounded organelle” (742 genes) and “intracellular anatomical structure” (867 genes). Within Reactome pathways, “metabolism of RNA” (93 genes) was the most significant term.

### 3.5. Differential Gene Expression and Enrichment in Thyroid Tissue under Cold Conditions

A total of 1149 upregulated BP terms were found (Table S4). Immune defense terms were seen as most significant, such as “immune response”, “regulation of immune system process” and “defense response” (Figure 4B). Those were followed by terms regarding response to external changes, including: “response to external stimulus” (88 genes), “positive regulation of response to stimulus” (83 genes), and “response to stimulus” (186 genes). A good number of BP terms surrounding cell communication and signaling have been found, with terms such as “cell surface receptor signaling pathway” (98 genes), “signaling” (160 genes) and “signal transduction” (147 genes). Moreover, cell cycle and differentiation terms were significant, such as: “sister chromatid segregation” (13 genes), “regulation of cell activation” (36 genes) and “metaphase/anaphase transition of cell cycle” (11 genes). In addition, stress-related terms were present: “response to stress” (42 genes), “regulation of response to stress” (45 genes) and “cellular response to stress” (4 genes). The most significant MF terms were “extracellular matrix structural constituent” (20 genes), “immune receptor activity” (11 genes) and “signaling receptor activity” (44 genes). The most significant CC terms consisted of “extracellular matrix” (45 genes), “cell periphery” (180 genes) and “extracellular region” (124 genes). The most significant Reactome pathways found were “arachidonic acid metabolism” (6 genes) and “extracellular matrix organization” (19 genes).

A total of 1982 downregulated BP terms were found (Table S4). The most significant terms were involved in RNA synthesis and maturation, such as: “ribonucleoprotein complex biogenesis”, “ribosome biogenesis” and “RNA processing” (Figure 4B). These were closely followed by protein maturation-related terms, such as “response to unfolded protein” (70 genes), “protein localization” (534 genes) and “protein transport” (376 genes). The most significant terms for the MF category were “RNA binding” (449 genes), “protein binding” (2112 genes) and “ribonucleoprotein complex binding” (61 genes). The most significant terms for the CC group included “intracellular membrane-bounded organelle” (1916 genes), “cytoplasm” (1849 genes) and “intracellular anatomical structure” (2188 genes). Pathways related to proteins and ribosome processing were found through KEGG analysis: “protein processing in endoplasmic reticulum” (70 genes) and “ribosome biogenesis in eukaryotes” (29 genes), as well as through Reactome “rRNA modification in the nucleus and cytosol” (44 genes) and “metabolism of RNA” (231 genes).

### 3.6. Differential Gene Expression and Enrichment in Thyroid Tissue under Ambient-Temperature Conditions

A total of 968 upregulated BP terms were found (Table S4). Most significant terms regarded the cell cycle, such as “sister chromatid segregation”, “regulation of mitotic metaphase/anaphase transition” and “regulation of chromosome segregation” (Figure 5B). In addition, many terms involved in the immune defense appeared significant, such as “regulation of immune system process” (32 genes), “antiviral innate immune response” (6 genes) and “immune response” (76 genes). For the MF category, these terms were regarded as most significant: “extracellular matrix structural constituent” (24 genes), “microtubule binding” (20 genes) and “ATPase” (24 genes). Within the most significant terms of the CC group, “extracellular matrix” (40 genes), “cell periphery” (182 genes) and “chromosome, centromeric region” (9 genes) were annotated. The most significant Reactome pathways were “mitotic spindle checkpoint” (11 genes) and “interferon alpha/beta signaling” (4 genes).

A total of 1860 downregulated BP terms were found (Table S4). The most significant terms were involved in ribosome and protein synthesis and metabolism, with terms such as “ribonucleoprotein complex biogenesis”, “ribosome biogenesis” and “protein localization” (Figure 5B). These were followed by catabolism terms such as “regulation of catabolic process” (265 genes), “catabolic process” (558 genes) and “cellular catabolic process” (490 genes). Further, a couple of stress-related terms appeared significant: “cellular response to stress” (438 genes) and “response to stress” (179 genes). The most significant terms in the MF category were “RNA binding” (440 genes), “protein binding” (2280 genes) and “enzyme binding” (427 genes). For the CC group, “intracellular membrane-bounded organelle” (2065 genes), “intracellular anatomical structure” (2362 genes) and “cytoplasm” (1982 genes), were the most significant terms. The most significant KEGG pathways were “protein processing in endoplasmic reticulum” (52 genes) and “protein export” (12 genes), accompanied by “rRNA modification in the nucleus and cytosol” (40 genes) and “metabolism of RNA” (214 genes) by Reactome pathways.

### 3.7. RT-qPCR Validation of RNAseq Results, and Expression of Selected Genes Involved in Thermogenesis and BAT Whitening

The trend and magnitude of the fold change of expression (log2FC ± SE) detected in the RT-qPCR analysis for all the tested genes, was similar to that detected in RNAseq results. Therefore, the results from the validation through RT-qPCR (Figure S2) from selected genes confirmed the accuracy of the RNASeq data to quantify gene expression in BAT and thyroid tissue. Fold change values (RT-qPCR vs. RNAseq analysis) were analyzed in BAT (cold/control) for *VEGFA (*−3.20 ± 0.22 vs. −1.79 ± 0.42), *PPARGC1A (*−0.47 ± 0.15 vs. −0.52 ± 0.53), *KI67* (1.36 ± 0.25 vs. 2.39 ± 0.58), *BMP4* (1.28 ± 0.15 vs. 0.97 ± 0.40), *ADRB3* (−1.22 ± 0.28 vs. −1.23 ± 0.58) and *DIO2* (−0.82 ± 0.38 vs. −0.83 ± 1.15). Fold change values (RT-qPCR vs. RNAseq analysis) were analyzed in BAT (AT/control) for *VEGFA (*−3.02 ± 0.24 vs. −1.53 ± 0.42), *PPARGC1A (*−1.47 ± 0.18 vs. −1.30 ± 0.53), *KI67* (0.64 ± 0.22 vs. 1.45 ± 0.58), *BMP4* (0.86 ± 0.11 vs. 1.08 ± 0.40), *ADRB3* (−1.20 ± 0.28 vs. −0.87 ± 0.58) and *DIO2* (−1.97 ± 0.78 vs. −1.12 ± 1.15). Fold change values (RT-qPCR vs. RNAseq analysis) were analyzed in thyroid tissue (cold/control) for *VEGFA (*−1.78 ± 0.56 vs. −1.32 ± 0.32), *PPARGC1A (*−1.61 ± 0.07 vs. −1.23 ± 0.27), *KI67* (2.34 ± 0.17 vs. 3.01 ± 0.70), *BMP4* (0.73 ± 0.35 vs. 0.43 ± 0.52), *ADRB3* (−0.05 ± 0.56 vs. NA) and *DIO2* (−0.45 ± 0.12 vs. −1.23 ± 0.63). Fold change values (RT-qPCR vs. RNAseq analysis) were analyzed in thyroid tissue (AT/control) for *VEGFA (*−1.51 ± 0.34 vs. −1.16 ± 0.32), *PPARGC1A (*−1.40 ± 0.25 vs. −1.20 ± 0.27), *KI67* (1.59 ± 0.23 vs. 3.33 ± 0.70), *BMP4* (0.52 ± 0.15 vs. 1.23 ± 0.52), *ADRB3* (−0.53 ± 0.51 vs. NA) and *DIO2* (−0.75 ± 0.35 vs. −1.03 ± 0.63). There were not enough counts for the calculation of fold change values for *ADRB3* from RNAseq analysis in thyroid tissue, for neither the cold nor ambient-temperature groups.

**Figure 6:**
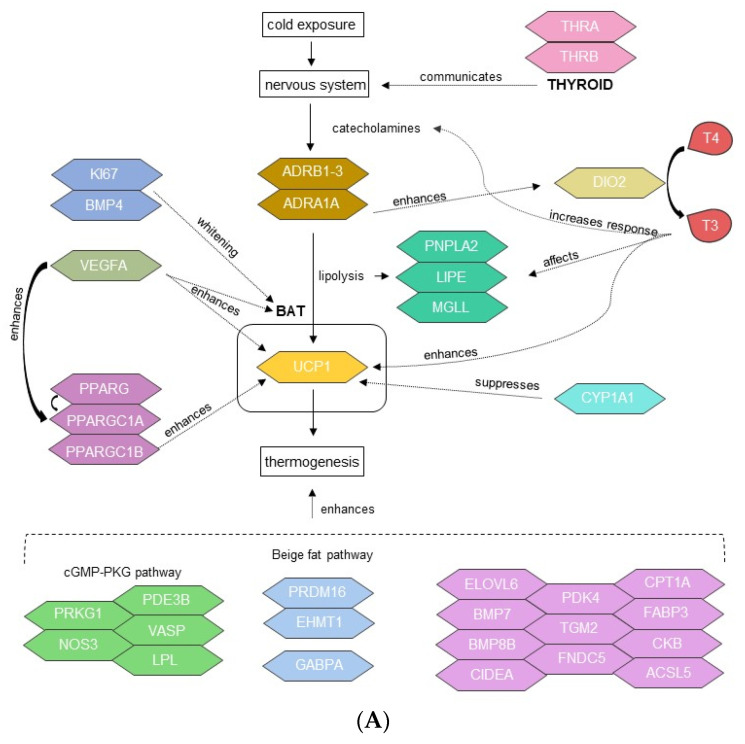

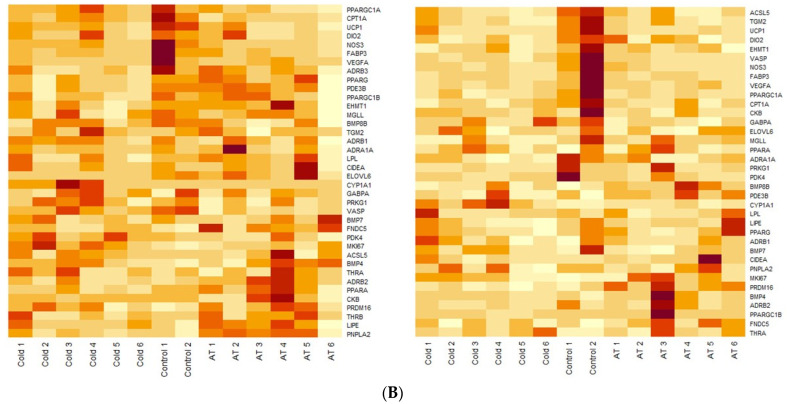
Characters involved in thermogenesis and BAT whitening. (**A**) Diagram of the thermogenic/whitening network, composed by the 38 analyzed genes. (**B**) Heatmaps of the 38 uniquely analyzed genes in BAT (left) and thyroid tissue (right), clustered from the normalized counts per gene matrix from the DESeq2 analysis for each group of samples; cold, control and ambient. The genes *ADRB3* and *THRB* were excluded from the thyroid heatmap due to insufficient counts. Cold 1 to Cold 6 represent animals kept at 4 °C (cold) for two days, AT 1 to AT 6 represent animals kept at 20–22 °C (ambient temperature) for two days, and Control 1 and Control 2 represent the control lambs that were euthanized within 12–24 h after birth.

A set of 38 genes (Table S3) which are known to have an active role directly or indirectly on thermogenesis and BAT whitening (Figure 6) were selected and analyzed in both tissues as cold temperature vs. control, ambient temperature vs. control and cold temperature vs. ambient temperature (Table 1). In the analysis of cold temperature vs. control, *FABP3*, *NOS3* and *VEGFA* were downregulated in BAT, whereas *CYP1A1* and *MKI67* where upregulated; meanwhile, in thyroid tissue *PDK4*, *TGM2*, *ASCL5*, *CTP1A*, *FABP3*, *NOS3*, *VASP* and *VEGFA* were downregulated, while *CYP1A1A* and *MKI67* were upregulated. In the case of ambient temperature vs. control, *CPT1A*, *FABP3*, *NOS3*, *VASP* and *VEGFA* were downregulated in BAT, whereas *FNDC5*, *PRDM16* and *CYP1A1A* where upregulated. In thyroid tissue, *TGM2*, *ACSL5*, *CPT1A*, *FABP3*, *NOS3*, *VASP* and *VEGFA* were downregulated, while *CYP1A1A* and *MKI67* were upregulated. In the cold temperature vs. ambient temperature comparison, only *CYP1A1A* was observed as upregulated in BAT. There were no significant differences recorded in any tissue/treatment for *UCP1*, *ADRB1-3*, *ADRA1A*, *PPARGC1B*, *PPARA*, *PPARG*, *ELOVL6*, *BMP7*, *BMP8B*, *CIDEA*, *CKB*, *PRKG1*, *PDE3B*, *LPL*, *EHMT1*, *GABPA*, *THRA*, *THRB*, *DIO2*, *PNPLA2*, *LIPE* and *MGLL*.

## 4. Discussion

The thermogenic capacity of BAT under cold exposure is a multistep process, regulated by a complex network of genes and metabolic pathways [30]. Over the first few days of life these factors decline [23], as BAT transforms into WAT and shivering becomes the main response to cold conditions [4]. In this context, transcriptome profiles from a comparative RNAseq analysis of newborn lambs under cold and ambient conditions provide insight into the molecular changes that occur over this period. As there is minimal information available to date regarding the molecular aspects of these transitions [3,30], this current approach adds an in-depth view of some of the major characters that take part directly or indirectly in thermoregulation and the fat-whitening transformation.

### 4.1. Analysis on the Main Factors That Regulate Thermogenesis

Exposure to cold leads to an expected cascade of events (Figure 6), starting from an increase in catecholamines such as norepinephrine that stimulate the various subtypes of β-adrenergic receptors (ADRBs) normally found on the surface of brown adipocytes in BAT [58,59,60]. More importantly, it stimulates the adrenergic-B3-receptor, which can activate lipolysis and release fatty acids in BAT [14,61,62]. The key thermogenic factor *UCP1* (uncoupling protein 1), then utilizes fatty acids to produce heat instead of ATP in cold conditions. All lambs under cold and ambient temperature conditions in the current study displayed no difference in the expression of the *UCP1* gene or any of the ADRBs (*ADRB1-3* and *ADRA1A*), at day three of age. This observation may support previous results that were found around this age by both Basse et al. [3] and Lomax et al. [63], where *UCP1* expression was not detected in lambs by day four or five of age, respectively. On the other hand, some studies have found *UCP1* expression at older ages in sheep or even at younger ages in goats. A recent study by Liu et al. [64] in newborn goats, after 24 h of cold exposure, found (through RNAseq) an increase of expression of several regulatory genes for thermoregulation in BAT such as *ADRRB1* and *PPARGC1A* (peroxisome proliferator-activated receptor gamma coactivator 1-alpha)*,* and in addition, observed an increase in the protein levels of *UCP1*. Furthermore, results from an RNAseq study on perirenal BAT of six-month-old lambs subjected to cold conditions for 25 days reported an expression of *UCP1* [65]. Another study using the same tissue from suckling lambs reported an expression of *UCP1* through RNAseq at 19−35 days of age [66]. Yuan et al. [67] reported different levels of expression of this gene from 2 to 12 months of age, where lower levels were found in superficial fat, and higher levels were seen in deep fat deposits. This observation by Yuan et al. [67] could explain in part why *UCP1* expression was not observed in this study on day three, due to the possibility that the samples utilized here were coming from the surface of perirenal BAT. Unfortunately, more information regarding on the differences of gene expression within different parts of the adipose tissue are lacking. Other studies on this aspect focused more on differences between visceral and subcutaneous adipose tissue, which seem to have an independent metabolic function [68]. Nevertheless, in the said studies there was no mention on the differences between sub-compartments of each tissue type. However, BAT is amongst the most vascularized tissues in the body [69], where there is a big interaction between adipocytes and vascular cells [70]. For instance, vascular cells can express *VEGFA* [71], which boosts its proliferation and survival [72], where an overexpression of *VEGFA* is also considered a key factor responsible for the thermogenic response of BAT to cold exposure [73]. Consequently, this interaction between vascular cells and adipocytes might have an impact on gene expression, where adipose tissue extracted from a more proximal location to the vascular vessels could provide different expression results, compared to the expression of a tissue extracted in a more distal location from the vessels.

The lack of differential expression of the B-adrenergic receptors in the present study could have impacted negatively by not inducing the expression of *UCP1* in BAT. In combination with these reports and the present study, it could be that the expression of *UCP1* comes and goes very rapidly during the first critical few days of life, but can also have a more prolonged and noticeable expression during cold conditions in older lambs. These discrepancies around the time where *UCP1* loses expression might also be caused by the different breeds used in thermogenesis studies, the techniques being used to measure the gene expression or other experimental environmental factors [66]. It could also be suggested that the conditions of the present study were not cold enough to induce a response. However, the enrichment analysis of the lambs of this study resulted in several Biological Process GO terms regarding response to stress, regulation of response to external stimulus, and cell communication and signaling, which were upregulated during cold conditions. These observations imply that the cold environment was in fact challenging the lambs, as has also been previously reported in mice indoors at 4 °C for three days [30], and in goats kept indoors at 6 °C for 24 h [64]; therefore, one might conclude that the environment was cold enough to make a physiological response. Consequently, it appears that there is a need for further studies to collect more information on the timing of *UCP1* expression.

The absence of differential expression of *UCP1* was accompanied by a decline or lack of expression of genes primarily associated with BAT and thermoregulation, such as the PPARs (peroxisome proliferator-activated receptors), where some of the roles of this group of genes are the control of fatty acid oxidation [74] and adipogenesis [75], where their activation is subjected to transcriptional coactivation by *PPARGC1A* [6] and *PPARGC1B* (peroxisome proliferator-activated receptor gamma coactivator 1-beta) [76]. Thus, the lack of expression of *PPARA* or *PPARG* in this study was expected, as the expression of *PPARGC1A* was downregulated in thyroid tissue, and in BAT there was no difference of expression, under neither cold nor ambient conditions, plus there was no differential expression of *PPARGC1B* recorded in any tissue/treatment. *PPARGC1A* has been proposed to be a master regulator of BAT differentiation and also an inductor of the *UCP1* gene [6,77], hence making this gene indispensable for proper thermogenesis [78]. Consequently, the lack of expression of *PPARGC1A* in BAT and its downregulation in thyroid tissue could be impacting negatively on the thermogenic induction of *UCP1* and other thermogenic genes that it coactivates. Furthermore, there was a lack of expression in all tissues and treatments of several thermogenic genes that were previously reported to have an increased expression upon cold exposure. Genes regarded in those lines were: *ELOVL6* (Fatty Acid Elongase 6) as a regulator of fatty acid chain elongation [64,79]; *BMP7* (Bone Morphogenetic Protein 7), which promotes brown adipocyte differentiation [29,80]; *BMP8B* (Bone Morphogenetic Protein 8b) as an energy dissipator of BAT [81,82,83]; *CIDEA* (cell death-inducing DFFA-like effector A), which is an considered as a marker of BAT in rodents [84]; and *CKB* (Creatine Kinase B), from the creatine cycle that drives the thermogenic respiration in fat cells [85,86]. In addition, some genes related to thermogenesis were differentially expressed and even downregulated after cold exposure in some cases, but were not seen upregulated after cold exposure in any tissues (Table 1), such as BAT adipogenesis genes *PDK4* (Isozyme 4) [87], *TGM2* (Transglutaminase 2) [29] and *FNDC5* (Fibronectin Type III Domain Containing 5) [30]; *ACSL5* (Acyl CoA synthetase 5), which encodes long-chain acyl CoA synthetase, a key enzyme for β-oxidation [30]; *CPT1A* (Carnitine Palmitoyltransferase 1A), which is involved in the transport of fatty acids into the inner mitochondrial membrane for β-oxidation [88,89,90]; and *FABP3* (Fatty Acid Binding Protein 3), which is essential for accelerating fatty acid flux to its oxidation through *UCP1* [91].

According to previous reports, there are specific pathways which involve a group of genes with the principal objective to increase thermogenesis during cold adaptation. One of these pathways is the cGMP-PKG signaling pathway (Cyclic guanosine monophosphate—Protein Kinase G), which after activation through adrenergic receptors, can increase lipid uptake by BAT and further regulate thermogenesis [92,93,94]. This pathway involves genes such as *PRKG1* (Protein Kinase CGMP-Dependent 1)*, NOS3* (Nitric Oxide Synthase 3)*, PDE3B* (Phosphodiesterase 3B), *VASP* (Vasodilator Stimulated Phosphoprotein) and *LPL* (Lipoprotein Lipase), which were previously recorded as upregulated in perirenal BAT of newborn goats after cold exposure [64]. Nevertheless, the said genes were not upregulated in BAT or thyroid tissue after cold exposure in this study, and in some cases, they were downregulated (*NOS3* in BAT and thyroid, and *VASP* in thyroid). Additionally, other pathways have been described around autonomous ways to produce heat from beige fat, such as the *PRDM16* (PR domain containing 16) pathway, which together with the expression of *EHMT1* (Euchromatic Histone-Lysine N-Methyltransferase 1) can activate beige adipocytes biogenesis, enhancing adipose tissue thermogenesis [95,96,97]. Furthermore, another compensatory pathway of beige fat biogenesis has been described: the glycolytic beige fat, through the actions of *GABPA* (GA-Binding Protein alpha), which can be activated even in the absence of adrenergic receptor signaling [98]. However, neither of the genes described in these alternative thermogenic pathways were recorded as upregulated in BAT or thyroid tissue after cold exposure in our study. In summary, each of the genes described here, which were characterized as thermogenic and previously observed as activated after cold exposure, were not being positively expressed in any tissue after the lambs of this study were in a cold environment for two days. Moreover, there was a total lack of differential expression, either upregulated or downregulated, of any of those genes when comparing each tissue between the cold and ambient groups. Therefore, these results can help visualize the molecular state of BAT and thyroid tissue of newborn lambs exposed to cold, and how each and every character involved in thermogenesis is lacking its expression in order to produce heat in a non-shivering way.

One probable cause of this absence/loss of expression of these genes associated with thermoregulation might be the overall downregulation of *VEGFA* (vascular endothelium growth factor), as observed in both tissues and treatments in this study. Previous reports state that the overexpression of *VEGFA* promotes a “BAT-like” phenotype in the adipose tissue, and that it enhances the expression of BAT genes such as *PPARGC1A* and *UCP1* [99]. Further, *VEGFA* can stimulate adrenergic receptors, which consequently induce these thermogenic genes [73], since the ablation of *VEGFA* leads to a downregulation of B-adrenergic signaling to BAT [100]. Therefore, it might be expected that the downregulation of *VEGFA* could be a possible reason that the B-adrenergic receptors lacked expression, subsequently pulling down the expression of *PPARGC1A* and *UCP1*, thus reducing the thermogenic activity in these newborn lambs. Another possibility to explain the lack of expression of the key thermoregulator *UCP1* might be the overexpression of the *CYP1A1* gene (cytochrome P450 1A1), which was found in all tissues and treatments. The activity of the enzyme encoded by this gene can be stress-induced [101], which can be linked to the several BP GO terms regarding the response to stress that were seen in cold conditions, as previously described. Even though there were no records of stress-related terms in the lambs at ambient conditions, it could be presumed that there was some level of stress in these lambs due to the difference in temperature from birth. The ambient temperature utilized in this experiment is much lower than in the uterine environment, resulting in lambs needing to increase the rate of body heat production by up to fifteen times more than the fetal level to compensate for the heat loss [1,2]. Interestingly, this gene has been observed to be expressed in the inner mitochondrial membrane [102], exactly where *UPC1* acts producing heat, but in there *CYP1A1* releases arachidonic acid, which in turn suppresses the mitochondrial activity [103]. For this reason, it might be the case that the over-expression of this gene might cut down the processes to produce heat by *UCP1*, therefore impairing thermogenesis. Unfortunately, little is known about the molecular actions that involve this gene and how it interacts with other thermogenic genes; further work is required to understand this process.

### 4.2. BAT and Thyroid Thermogenesis Association

Thyroid hormones have a multifaceted contribution to thermogenesis, as there are about 8000 thyroid-hormone receptors per brown adipocyte cell [15]; hence, they would play an essential part in energy homeostasis during cold exposure as they activate heat production [16]. However, evidence of thermogenic activity was not observed in the present study as previously discussed; thus, the lack of differential expression in main thyroidal genes was not unexpected. Thyroid hormone receptor A (*THRA*), which mediates the communication between thyroid hormone signaling and the sympathetic nervous system in BAT, was found not to be differentially expressed in all tissues and treatments, as well as the receptor B (*THRB*), which mediates the tri-iodothyronine hormone (T3) regulation of *UCP1* in BAT [16]. Consequently, the lack of differential expression of these thyroid receptors suggests that there was neither induction of BAT through the hypothalamic pathway nor stimulation of the expression of *UCP1* by them. This missing thermogenic response has been previously observed in many mammals, including hypo-thyroidal newborn lambs [104], newborn calves [105,106,107] and piglets [108]. In these studies it has been shown that an impairment in heat production occurs as BAT becomes unresponsive to noradrenaline stimulation in the absence of thyroidal hormones [109]. Furthermore, the type II iodothyronine deiodinase enzyme (*DIO2*) was not differentially expressed in all tissues and treatments in the present study. This enzyme is responsible for the conversion of the inactive hormone, thyroxine (T4) to T3, which is the metabolically active form [14]. If active, DIO2 would have increased the catecholamine response and *UCP1* expression [19,20], but in the present study these actions were not observed. In fact, a study on *DIO2* knockout mice embryos showed an impairment in thermogenesis, which was associated with decreased expression of *UCP1* and *PPARGC1A* [110]. It could have been the case that the lack of expression of *ADRB3* found in the present study had a negative impact on *DIO2*, as it has been reported that the expression of *ADRB3* during cold exposure can stimulate the hormonal conversion of T4 to active T3 of *DIO2* [9,111]. Moreover, in a study in humans, Kurylowicz et al. [62] observed that a decrease in expression of *DIO2* resulted in a lower local conversion of T4 to T3, suggesting that those factors contributed to the reduced expression of *THRA* and *THRB* found in their study. These previous observations support the present study, where the importance of *DIO2* in heat production appears to be significant and it could have implications in the missing expression of *THRA* and *THRB* as found in this study. In summary, the possible absence of T3 production may have negatively affected lipolysis, as reported by both De Jesus et al. [111] and Thrush et al. [112]. This observation could support this study, since a differential expression of the genes that encode lipases were missing in all tissues and treatments (*PNPLA2* (patatin-like phospholipase domain containing 2), *LIPE* (lipase E) and *MGLL* (monoglyceride lipase)). Given that these genes were not apparently active, a reduced availability of fatty acids might have occurred, therefore decreasing the possibility for heat production due to a lack of a suitable “fuel” for the *UCP1* machinery.

### 4.3. Transition from BAT to WAT

Within a matter of days after birth, the majority of BAT present in large mammals commences its transformation into WAT and shivering thermogenesis becomes the main response to cold exposure [4,24]. This transition concludes with the loss of BAT and its thermogenic activity, where the B-adrenergic signaling has been found to be diminished [100,113]. This latter observation may be in accordance with the present results and places the lambs at the beginning of this period, as there were no differences found in the expression of any of the ADRBs analyzed (*ADRB1-3*) between treatments. Further, this lack of sensitivity to the adrenergic pathways seems to be interconnected with the lack of or missing expression of key thermogenic genes found here, such as *UCP1* and *DIO2*, and its transcription activators (PPARs) that were found to be not differentially expressed in the present study’s lambs, or even downregulated as *PPARGC1A* in thyroid tissue in all lambs. Basse et al. [3], utilizing RNAseq in newborn lambs’ perirenal adipose tissue, found not only the loss of *UCP1*, but also the loss of BAT-enriched factors such as *PPARGC1A*, *PPARG* and *DIO2*, which were reduced by day one and poorly expressed after day four. Moreover, another study [63] observed that the sharp fall of *UCP1* on the first day of life was also accompanied by the decline of *PPARGC1A* and *PPARA*, even when the lambs were maintained below their thermoneutral zone. Lomax et al. [63], showed that this cascade of lack or even loss of expression in these key BAT markers occurs soon after birth and marks the ontogenically programmed transformation of BAT to WAT.

Another probable leading factor in this transformation could be the overall underexpression of *VEGFA*. The downregulation of this gene could have imposed an opposite “BAT-like” phenotype in all lambs in this study, which impeded thermogenesis activation. Shimizu et al. [100] observed that *VEGFA* knockout mice had lipid accumulation and mitochondrial dysfunction in adipose tissues, accompanied by an impairment of noradrenergic signaling, suggesting that the absence or decrease in this gene is a primary factor that leads to BAT whitening. Additionally, Kotzbeck et al. [114] stated that BAT whitening is not only induced by factors such as β-adrenergic signaling impairment, but to a lipase deficiency. Therefore, each of these factors, as the lack of differential expression of the ADRBs and lipases (*PNPLA2*, *LIPE*, *MGLL*) found in all lambs in the study between treatments, is capable of inducing macrophage infiltration and brown adipocyte death [114]. In addition, *VEGFA* has anti-inflammatory properties in the adipose tissue [99]; consequently, the reduction in its expression could be leaving BAT unprotected from the inflammation processes that takes place in its transformation to WAT. Consequently, this differentiation from brown to white adipocytes would be initiated, which could also be implied by the overexpression of *KI67* (monoclonal antibody KI67) found in all lamb tissues and treatments and the overexpression of *BMP4* (bone morphogenetic protein 4) found in both tissues under ambient conditions. It is known that these two genes are involved in WAT adipogenesis, where a rise in *BMP4* expression was previously observed with the loss of the BAT phenotype and the continuing differentiation of white adipocytes [29], implying that it might be marking the start of a change over to a WAT phenotype. Pope et al. [29] also found an overexpression of *KI67* in the proliferative state of white adipocytes, where *UCP1* was not differentially expressed. The present results seem to be in compliance with Pope et al.’s study, where the overexpression of key WAT genes mix in with the lack of differential expression of *UCP1* found here, resulting in the possible ending of BAT and the non-shivering thermogenesis period.

### 4.4. Downregulation of Protein Synthesis

Gene Ontology and pathway analysis showed that the majority of downregulated DEGs in the present tissues and treatments were enriched towards ribosome and protein synthesis and maturation terms. It is known that protein synthesis is energetically expensive [115] and under stress conditions, a rapid attenuation or even a global shutdown of protein synthesis has been described [116]. Since protein misfolding sets a major risk to health of cells and organisms, a protein quality control mechanism exists to maintain protein homeostasis [117,118,119]. Liu et al. [116] observed that this quality control responds under adverse conditions with an inhibition of the translation initiation by halting ribosomes during early elongation. This pausing could represent a co-translational stress response in order to maintain intracellular protein homeostasis, while adapting to a change in the environmental conditions [116,120]. It might be expected to find this downregulation of protein synthesis in both tissues under cold conditions in the present study, as the lambs were subjected to cold and required energy expenditure (heat production) to thermoregulate. This halt in protein production was observed in both tissues under ambient conditions; however, it is possible that the lambs subjected to ambient conditions in the present study could also be experiencing some level of thermoregulatory stress and reconditioning after leaving the uterine environment. Therefore, it could be supposed that all lambs regardless of treatment might be experiencing decreasing protein production as a transient response to the differential environmental conditions post birth. It would be of interest in further studies to determine how long post-birth this activity occurs.

### 4.5. Highly Expressed Immune Defence and Cell Cycle Processes

Gene Ontology and pathway analysis also revealed that the majority of the upregulated DEGs in both tissue types and lamb treatments, were enriched towards immune defense response and cell cycle terms. These immune defense process findings are expected, as it is known that the days following birth are a very vulnerable period of life. The development of the immune system commences during the early stages of fetal life, but at birth, newborn lambs are immunologically naïve [121], as immunoglobulins are not passed through the ovine placenta [122,123]. Passive immunity is provided via colostrum intake, but as observed by Sanglid et al., [124], the newborn’s gut needs to be permeable to the passage of macromolecules, therefore leaving the lamb vulnerable to pathogen ingestion. In accordance with the multiple GO terms and pathways found, including those classified as being “defense response”, “regulation of immune system process” and “response to virus”, the lambs in the present study had their immune defense boosted above most other biological processes. This biological process likely helps prepare the lambs against possible viral infections and other harmful pathogens. In relation to the increase in cell cycle terms, this abundance in cell proliferation may come from the postnatal transformation of brown adipocytes to white adipocytes. Basse et al. [3], who observed the transition from BAT to WAT in sheep, stated that it could be the case that the proliferation of white adipose precursor cells was in fact contributing to whitening. This statement potentially supports the idea that the rise in cell proliferation seen in the samples of this study might be linked to the whitening process, as this seems to be added as part of that cascade of events, which was previously discussed here.

## 5. Conclusions

This study provides an in-depth gene expression analysis of the main characters involved in the thermogenesis and whitening mechanisms that take place in the newborn lamb. The data from the present study add value to the understanding of the molecular processes that underlie these changes in first few days of life where currently little is known, and the interaction between these complex factors. This study shows that the heat production peak under cold exposure occurs in a very fast and immediate way, such that it may seem undetectable by day 3 of life. Moreover, these changes in expression might give way to the whitening of the adipose tissue, summing up the non-shivering thermogenesis period.

## Figures and Tables

**Table 1:** Selected genes with a role in thermoregulation or BAT whitening within each group.

Gene	Tissue	Cold/Ctrl log2FoldChange	Cold/Ctrl *p*-adj	AT/Ctrl log2FoldChange	AT/Ctrl *p*-adj	Cold/AT log2FoldChange	Cold/AT *p*-adj
*PPARGC1A*	BAT	−0.52	0.64	−1.3	0.08	0.78	0.32
	Thyroid	−1.23	<0.05	−1.2	<0.05	−0.03	1.00
*BMP4*	BAT	0.97	0.13	1.08	<0.05	−0.11	0.89
	Thyroid	0.43	0.59	1.23	0.06	−0.80	0.96
*PDK4*	BAT	0.76	0.48	−0.29	0.79	1.04	0.17
	Thyroid	−2.40	<0.05	−1.83	0.09	−0.57	1.00
*TGM2*	BAT	0.14	0.85	−0.05	0.94	0.19	0.72
	Thyroid	−1.36	<0.05	−1.31	<0.05	−0.05	1.00
*FNDC5*	BAT	1.27	0.06	1.95	<0.05	−0.69	0.27
	Thyroid	0.33	0.65	0.97	0.11	−0.64	1.00
*ACSL5*	BAT	0.28	0.73	0.28	0.65	−0.01	0.99
	Thyroid	−0.88	<0.05	−0.81	<0.05	−0.07	1.00
*CPT1A*	BAT	−0.95	0.06	−1.59	<0.05	0.63	0.17
	Thyroid	−0.94	<0.05	−0.93	<0.05	0.00	1.00
*FABP3*	BAT	−1.74	<0.05	−2.01	<0.05	0.27	0.78
	Thyroid	−2.20	<0.05	−2.29	<0.05	0.09	1.00
*NOS3*	BAT	−1.40	<0.05	−1.87	<0.05	0.47	0.35
	Thyroid	−1.53	<0.05	−1.46	<0.05	−0.07	1.00
*VASP*	BAT	−0.38	0.33	−0.75	<0.05	0.37	0.23
	Thyroid	−0.94	<0.05	−0.92	<0.05	−0.02	1.00
*PRDM16*	BAT	0.98	0.15	1.16	<0.05	−0.18	0.81
	Thyroid	−0.12	0.87	0.72	0.21	−0.85	0.66
*VEGFA*	BAT	−1.79	<0.05	−1.53	<0.05	−0.26	0.73
	Thyroid	−1.32	<0.05	−1.16	<0.05	−0.16	1.00
*CYP1A1*	BAT	12.80	<0.05	7.33	<0.05	5.47	<0.05
	Thyroid	8.98	<0.05	6.44	<0.05	2.54	0.22
*MKI67*	BAT	2.39	<0.05	1.45	0.07	0.94	0.25
	Thyroid	3.01	<0.05	3.33	<0.05	−0.33	1.00

*UCP1*, *ADRB1-3*, *ADRA1A*, *PPARGC1B*, *PPARA*, *PPARG*, *ELOVL6*, *BMP7*, *BMP8B*, *CIDEA*, *CKB*, *PRKG1*, *PDE3B*, *LPL*, *EHMT1*, *GABPA*, *THRA*, *THRB*, *DIO2*, *PNPLA2*, *LIPE* and *MGLL* were not included in this table as they were not significant in any tissue/treatment (*p*-adj > 0.05). Cold/Ctrl: analysis of cold temperature vs. control; AT/Ctrl: analysis of ambient temperature vs. control; Cold/AT: analysis of cold temperature vs. ambient temperature.

## Data Availability

The dataset supporting the conclusions of this article is available in the NCBI Gene Expression Omnibus (GEO accession number: GSE210259, https://www.ncbi.nlm.nih.gov/geo/query/acc.cgi?acc=GSE210259).

## Supplementary Materials

The following supporting information can be downloaded at: https://www.mdpi.com/article/10.3390/metabo12100996/s1, Table S1: Read numbers throughout the analysis and RIN values of RNA samples; Table S2: DEGs list per tissue-treatment; Table S3: DESeq results for the 38 analyzed genes; Table S4: GO terms and pathways per tissue-treatment; Table S5: Primer sequences for RT-qPCR; Figure S1: PCA plots of control vs. cold vs. ambient-temperature groups of BAT and thyroid tissue; Figure S2: RT-qPCR validation figures.

## Abbreviations

*UCP1*: uncoupling protein 1; *ACTB*: Beta-actin; *ADRB1-3*: adrenoceptor beta 1-3; *ADRA1A*: Adrenoceptor Alpha 1A; *BMP4*: bone morphogenetic protein 4; *CYP1A1*: cytochrome P450 1A1; *DIO2*: iodothyronine deiodinase 2; *GAPDH*: Glyceraldehyde-3-phosphate dehydrogenase; *HUWE1*: HECT, UBA and WWE domain-containing 1; *KI67*: monoclonal antibody KI67; *LIPE*: lipase E; *MGLL*: monoglyceride lipase; *PPARGC1A*: peroxisome proliferator-activated receptor gamma coactivator 1-alpha; *PPARGC1B*: peroxisome proliferator-activated receptor gamma coactivator 1-beta; *PNPLA2*: patatin-like phospholipase domain-containing 2; *PPARA*: peroxisome proliferator activated receptor alpha; *PPARG*: peroxisome proliferator activated receptor gamma; *RPL19*: ribosomal protein L19; *THRA*: thyroid hormone receptor alpha; *THRB*: thyroid hormone receptor beta; T3: tri-iodothyronine; T4: thyroxine; *VEGFA*: vascular endothelium growth factor; *ELOVL6*: Fatty Acid Elongase 6; *BMP7*: Bone Morphogenetic Protein 7; *BMP8B*: Bone Morphogenetic Protein 8b; *CIDEA*: Cell Death-inducing DFFA-like Effector A; *CKB*: Creatine Kinase B; *PDK4*: Isozyme 4; *TGM2*: Transglutaminase 2; *FNDC5*: Fibronectin Type III Domain Containing 5; *ACSL5*: Acyl CoA synthetase 5; *CPT1A*: Carnitine Palmitoyltransferase 1A; *FABP3*: Fatty Acid-Binding Protein 3; *PRKG1*: Protein Kinase CGMP-Dependent 1; *NOS3*: Nitric Oxide Synthase 3; *PDE3B*: Phosphodiesterase 3B; *VASP*: Vasodilator Stimulated Phosphoprotein; *LPL*: Lipoprotein Lipase; *PRDM16*: PR domain-containing 16; *EHMT1*: Euchromatic Histone-Lysine N-Methyltransferase 1; *GABPA*: GA-Binding Protein alpha.
