# Supplementary material for: RNAseq Analysis of Brown Adipose Tissue and Thyroid of Newborn Lambs Subjected to Short-Term Cold Exposure Reveals Signs of Early Whitening of Adipose Tissue
Source: Metabolites. 2022 Oct 20;12(10):996. doi: 10.3390/metabo12100996 (PMC9607389; doi:10.3390/metabo12100996)

Figure S1. Principal component analysis (PCA) plots of normalised sequence read counts in control vs. cold vs. ambient temperature groups for brown adipose tissue (BAT) (left) and thyroid tissue (TH) (right).

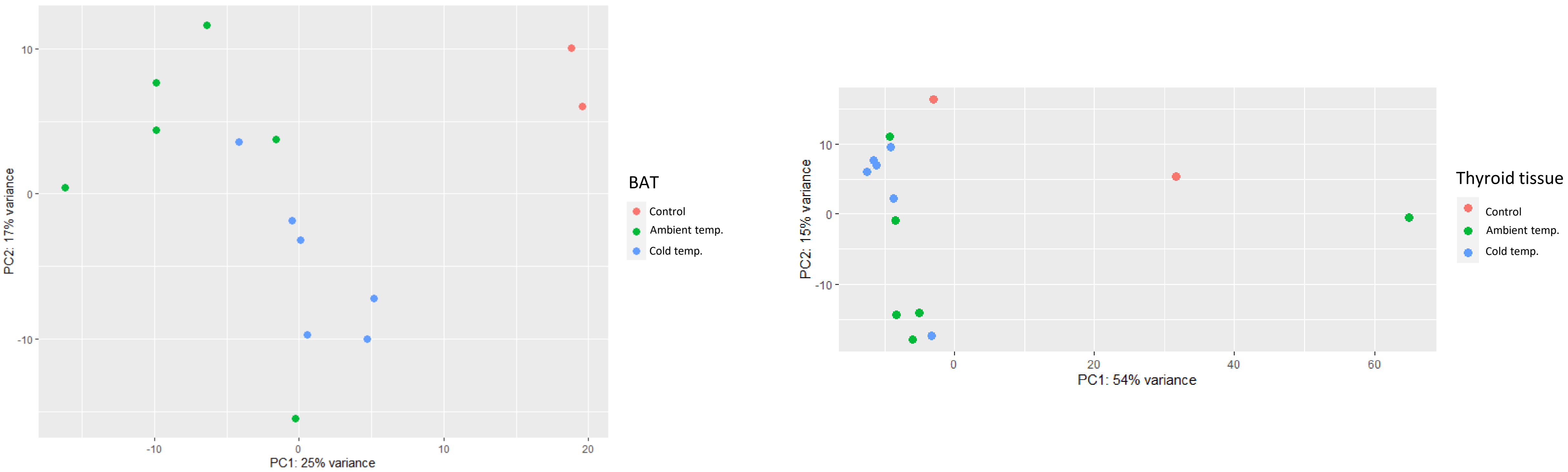

Supplement: Supplementary file 1 [file metabolites-12-00996-s001.zip › Additional file 4. Figure S1. PCA plots of control vs. cold vs. ambient temperature groups of BAT and thyroid tissue.pdf]
