# Supplementary material for: RNAseq Analysis of Brown Adipose Tissue and Thyroid of Newborn Lambs Subjected to Short-Term Cold Exposure Reveals Signs of Early Whitening of Adipose Tissue
Source: Metabolites. 2022 Oct 20;12(10):996. doi: 10.3390/metabo12100996 (PMC9607389; doi:10.3390/metabo12100996)
Supplement: Supplementary file 1 [file metabolites-12-00996-s001.zip › Additional file 7. Figure S2. RT-qPCR validation figures.pdf]

Figure S2. Fold change results of RT-qPCR target and endogenous genes compared to its corresponding RNAseq result (log2FC ± SE).

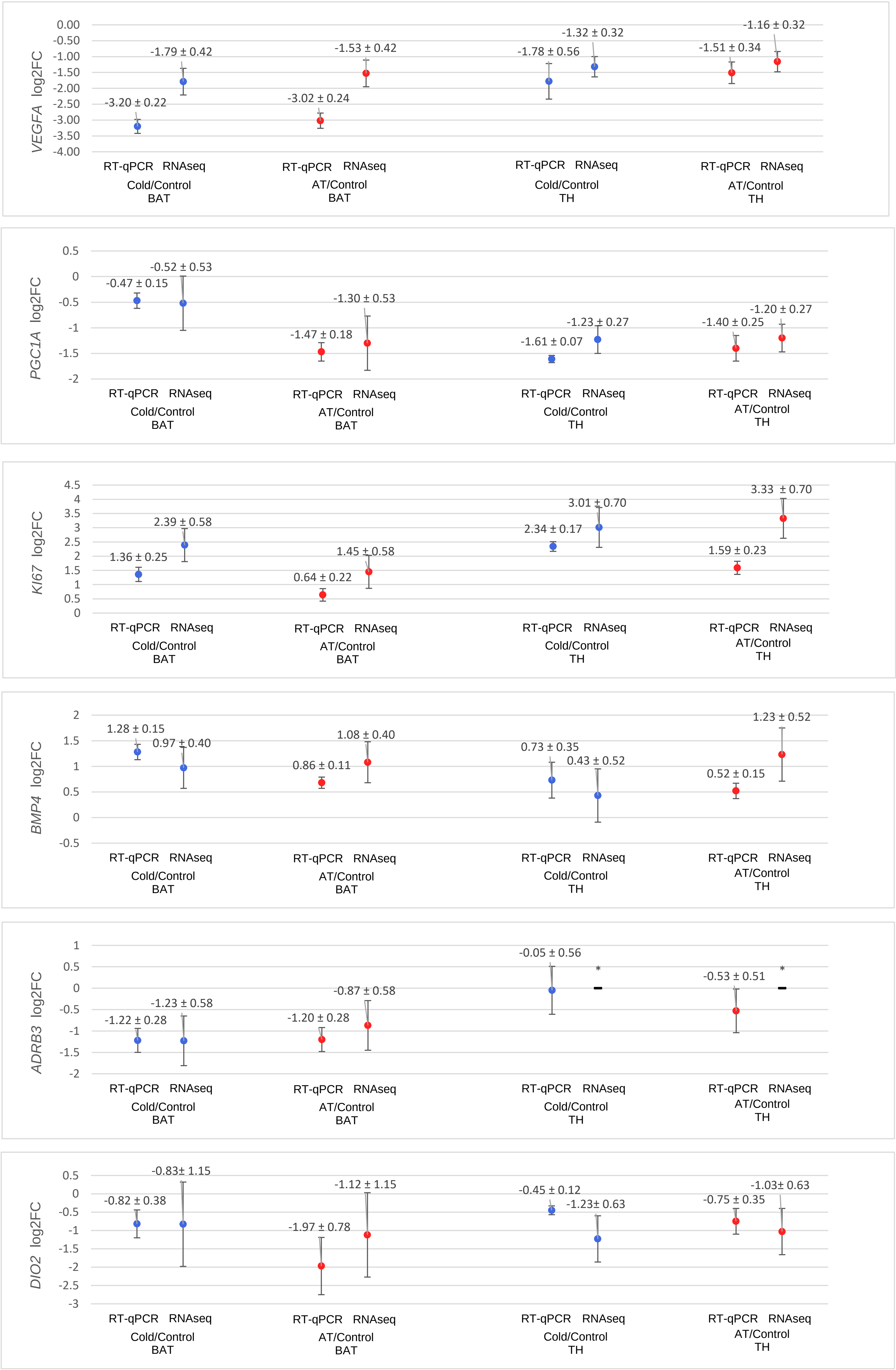

* Not enough counts for calculation of log2FC of *ADRB3* in thyroid tissue via RNAseq
